# Giant Subcutaneous Leiomyosarcoma of Anterior Abdominal Wall

**DOI:** 10.1155/2014/308916

**Published:** 2014-11-18

**Authors:** Sanghamitra Jena, Samir Bhattacharya, Shravasti Roy

**Affiliations:** ^1^Department of Surgical Oncology, Saroj Gupta Cancer Centre and Research Institute, Mahatma Gandhi Road, Thakurpukur, Kolkata 700063, India; ^2^Department of Pathology, Saroj Gupta Cancer Centre and Research Institute, Mahatma Gandhi Road, Thakurpukur, Kolkata 700063, India

## Abstract

Subcutaneous leiomyosarcomas are rare tumors accounting for 1% to 2% of all superficial soft tissue malignancies. Although they may arise anywhere in the body, they most frequently occur in the lower extremities. The incidence of subcutaneous LMS affecting the anterior abdominal wall is very rare. We herein report the case of a patient with a giant subcutaneous leiomyosarcoma arising in the anterior abdominal wall. It was diagnosed by histopathology and immunohistochemistry and treated accordingly.

## 1. Introduction

Sarcomas of the soft tissue are a very rare condition, comprising approximately 1% of malignant tumors [1]. Leiomyosarcomas (LMS) are rare aggressive mesenchymal neoplasms, which arise from smooth muscles. They account for 3–7% of soft tissue sarcomas [2]. LMSs are divided into those involving deep soft tissues sites as the retroperitoneum and those involving peripheral soft tissue sites. These peripheral soft tissue LMSs, also referred to as superficial LMSs, are divided into cutaneous and subcutaneous LMSs on the basis of their histogenesis and different clinical and prognostic implications [3].

Subcutaneous LMS is thought to arise from small- to medium-sized blood vessels in the subcutaneous tissue [4]. It accounts for 1-2% of all soft tissue sarcomas [5]. Although they may arise anywhere in the body [6], they most frequently occur in the lower extremities [6]. The thigh has been reported as the most frequently affected site [7]. Only 10–15% of subcutaneous leiomyosarcomas arise in the trunk [8]. The incidence of subcutaneous LMS affecting the anterior abdominal wall is very rare [6]. We herein report the case of a patient with a giant subcutaneous leiomyosarcoma arising in the anterior abdominal wall.

## 2. Case Report

A 35-year-old female presented with an asymmetric, voluminous tumor on the right side of her abdomen, which had gradually grown during the past one year. The patient had three children and had no family history of neoplasia. She was not addicted to tobacco and alcohol. She had a history of surgical excision of a solid tumor of size about 3 cm × 5 cm, located in the right hypochondrium. The surgery was done two years back. She had biopsy report of spindle cell neoplasm, but no slides or blocks were available for review at our institute. The surgery was not followed by radiotherapy or chemotherapy.

**Figure 1:**
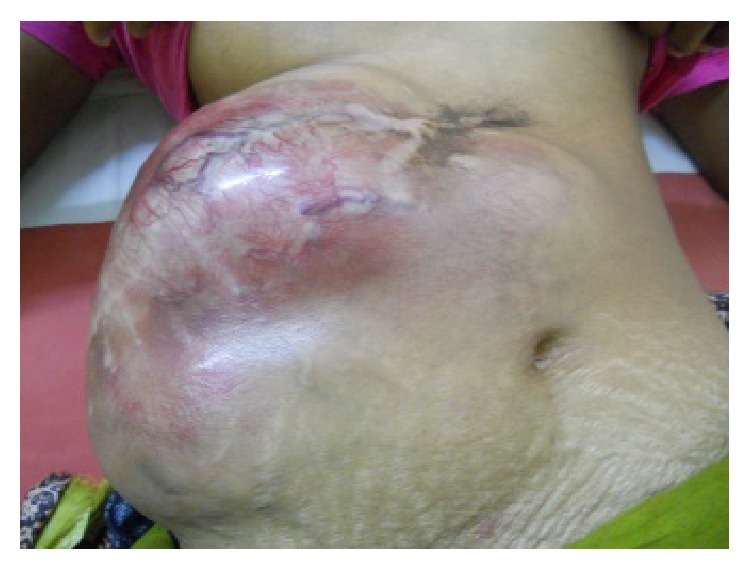
Clinical photograph of the patient.

Physical examination on admission revealed a tumor of size around 20 cm × 20 cm in the right side of abdomen, extending to the opposite side beyond the umbilicus (Figure 1). The tumor was fixed to the overlying skin and the underlying muscle. The skin appeared reddish with vascular engorgement, but there was no ulceration. It was not accompanied with any regional lymphadenopathy.

**Figure 2:**
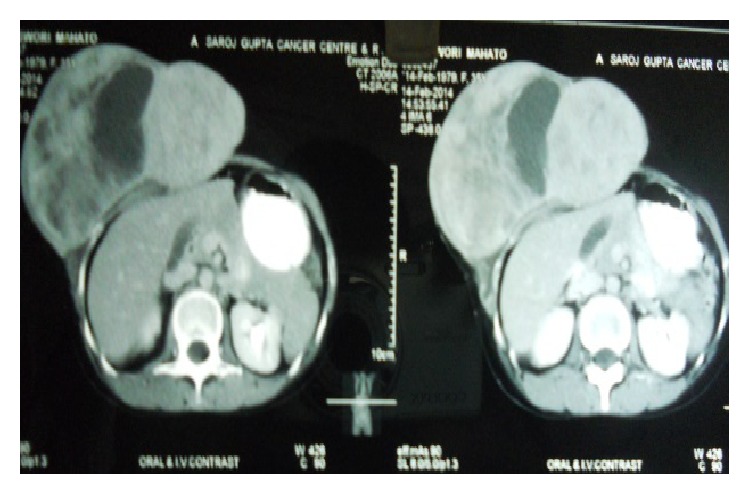
CT scan of the lesion.

Computed tomography (CT scan) depicted a large lobulated heterogeneously contrast enhancing soft tissue mass on the right side of anterior abdominal wall, extending from the lower chest wall downwards up to right side of pelvis, inseparable from right rectus, external oblique, and internal oblique muscles (Figure 2). There was no evidence of metastases to distant organs on imaging. Fine needle aspiration cytology of the lesion showed plump spindle cells in clusters and small groups, suggesting recurrent spindle cell neoplasm.

**Figure 3:**
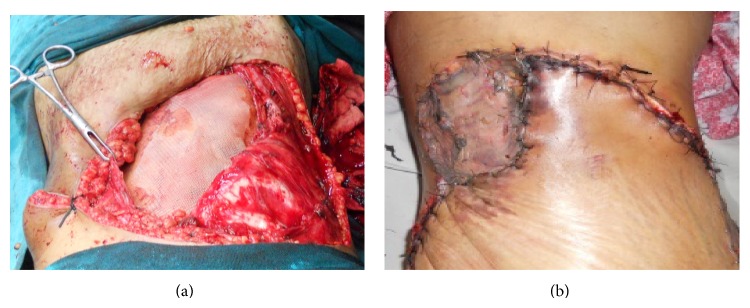
(a) Intraoperative photograph showing repair with prolene mesh. (b) Photograph showing skin defect coverage with rotational skin flap and split skin grafting.

She was subjected to surgery which consisted of a wide resection of the tumor along with the surrounding muscular layer and peritoneum. The resection exceeded the macroscopic limits of the tumor by 4 to 5 cm in order to accomplish a tumor-free margin. The defect was reconstructed with omentum and prolene mesh. The skin defect was covered with rotational skin flaps and split skin grafting (Figures 3(a) and 3(b)).

**Figure 4:**
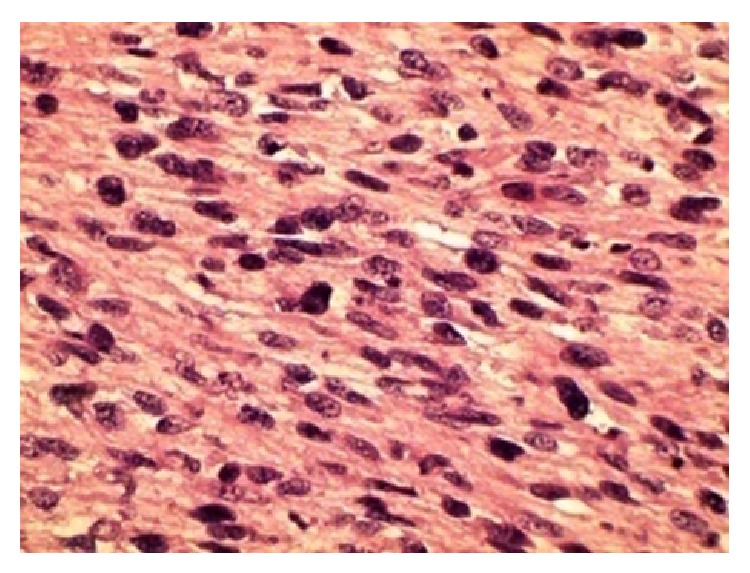
Histological sample of excised specimen (haematoxylin-eosin stain 40x).

**Figure 5:**
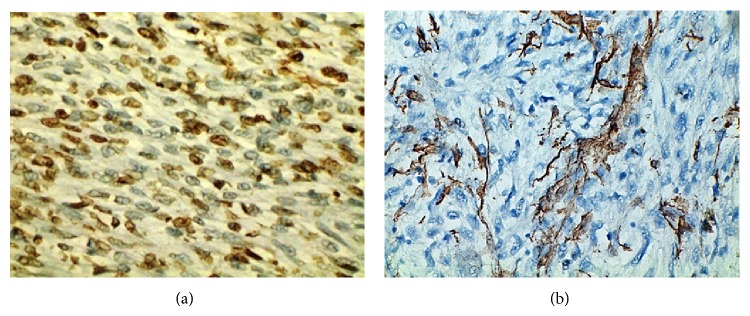
(a) Tumor cells positive for SMA (40x). (b) Tumor cells showing cytoplasmic positivity for SMA (40x).

The resected tumor, measuring 19 × 19 × 5 cm, was yellowish white in colour without central necrosis and hemorrhage on its cut surface. Routine histological examination with hematoxylin and eosin revealed that the tumor is comprised of spindle shaped cells arranged in sheets and fasciculated bundles with cigar shaped nuclei (Figure 4). Occasionally tumor giant cells were found. Mitotic figures were quite frequent, around 8–10 per 10 high-power fields. All the margins were free. In immunohistochemistry (IHC), the tumor cells were positive for smooth muscle actin (SMA) and CD34 but negative for desmin and S-100 (Figures 5(a) and 5(b)). The Ki-67 index was 10–15%. The histologic grade of the tumor (grading of the French Federation of Cancer Centers—FFCC) was Grade II (2 + 2 + 0). With these findings, the diagnosis of subcutaneous leiomyosarcoma was confirmed.

**Figure 6:**
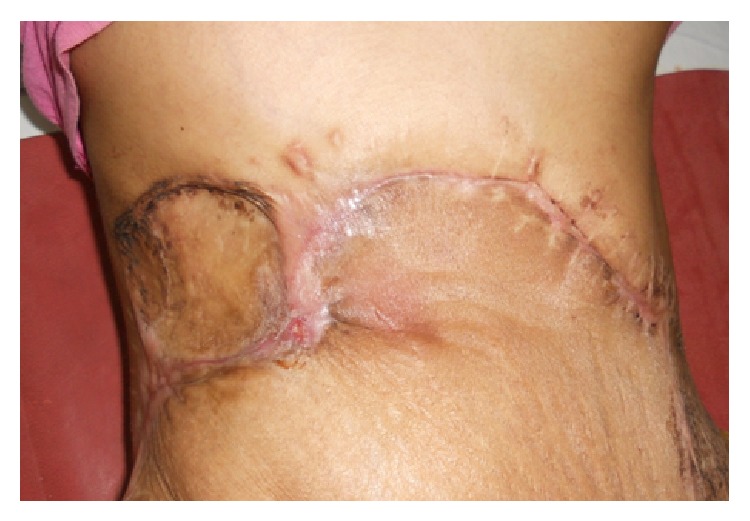
Postoperative photograph.

Postoperative period was uneventful (Figure 6). Taking into consideration the large tumor size, high mitotic rate, and recurrent setting, postoperative radiotherapy was given. The oncological treatment has now been in course for 4 months, with no clinical sign of local recurrence.

## 3. Discussion

Subcutaneous leiomyosarcomas are rare tumors accounting for 1% to 2% of all superficial soft tissue malignancies [5]. It usually occurs in patients between 50 and 70 years of age [6, 7], with a male predominance ranging from 2 : 1 to 3 : 1 [3, 6]. Although most tumors present as a subcutaneous nodule in the extremities, usually measuring 30 mm or less in diameter, about 10% of cases arise in the trunk [3, 6, 7, 9]. The incidence of subcutaneous LMS affecting the anterior abdominal wall is very rare [6]. Herein we have reported a case of a 35-year-old female presenting with a large (19 × 19 × 5 cm) anterior abdominal wall subcutaneous LMS.

Histologically, the tumor is characterized by the presence of perpendicularly arranged fascicles of spindled cells, with abundant eosinophilic cytoplasm that is dense but vacuolated and elongated blunt ended (“cigar shaped”) nuclei [3]. Immunohistochemical analysis plays a key role in differentiating leiomyosarcomas from other spindle cell tumors [4, 10]. The smooth muscle actin (SMA) is the most sensitive marker [3] and has been reported to be positive in 100% of the cases in many series of leiomyosarcomas [11]. In this patient, the characteristic histological features and positivity for SMA helped to reach the final diagnosis.

Surgical excision is the primary treatment for subcutaneous leiomyosarcoma. Until now, wide local excision, with 3 to 5 cm surgical margins, has been recommended, but recent literature has demonstrated the successful treatment for subcutaneous leiomyosarcoma with narrow margin excision as well as with Mohs micrographic excision [10]. Depending on the recurrent presentation of tumor in this case, we went for surgical excision with 3–5 cm margin.

Reconstruction of the abdominal wall after a wide resection can be accomplished either by using plastic surgery techniques, such as myocutaneous innervated free flaps [12], or by prosthetic reconstruction techniques [13]. The latter was chosen due to the fact that it allows a more extensive resection; furthermore, it does not require laborious manoeuvres involving blood vessels and nerves. Reconstruction with prosthetic mesh and cutaneous flaps is also a good option in cases where facilities for microvascular surgery and free flap reconstruction are not available.

Factors that have correlated with adverse prognosis in patients with subcutaneous leiomyosarcoma include tumor size, high mitotic rate, presence of necrosis, deep-seated tumors with fascial involvement, and intratumoral vascular invasion [8].

The role of adjuvant chemotherapy and radiation therapy in patients with superficial leiomyosarcoma is controversial [4]. Adjuvant radiotherapy may be reserved for tumors larger than 5 cm, for high grade tumors in order to reduce the likelihood of recurrence and in combination with surgery in cases of recurrent tumors [10].

Inadequate excision or local excision without adequate margin leads to recurrence and increases the risk for the metastatic and possibly fatal disease. Local recurrence may occur in 40% to 60% of patients, followed by distant metastases in 20% to 40% of cases [6, 7]. So long-term follow-up of the patients is essential.

## 4. Conclusion

Subcutaneous leiomyosarcoma is a rare clinical entity. Presence of a large anterior abdominal wall tumor may lead to diagnosis of soft tissue sarcoma other than subcutaneous LMS, which is very rare at this site. So this case report should increase awareness about the unusual presentation of subcutaneous LMS and help us in guiding how to manage such surgical defects, in places where facilities for microvascular surgery and free flap reconstruction are not available.
